# Association analysis between vitamin D level and depression in women perimenopause: A protocol of systematic review and meta-analysis

**DOI:** 10.1097/MD.0000000000020416

**Published:** 2020-05-22

**Authors:** Jie Yuan, Tiantian Chen, Yaling Lei, Shujun Wei, Penglong Yu, Yue Cao, Yuan Zhao, Jie Chen

**Affiliations:** a School of Basic Medical Sciences, Chengdu University of Traditional Chinese Medicine; b Department of Encephalopathy, Shaanxi Provincial Hospital of Traditional Chinese Medicine, Xi’an; c Department of Rheumatology, Hospital of Chengdu University of Traditional Chinese Medicine, Chengdu, P.R. China.

**Keywords:** meta-analysis, perimenopausal depression, protocol, vitamin D level

## Abstract

**Background:**

In recent decades, many researches manifested that the perimenopause is a window of vulnerability for the development of both depressive symptoms and major depressive episodes. Some scholar thought that those women diagnosed with depression may be particularly sensitive to changes in the hormonal milieu experienced premenstrual, during the postpartum period or during the menopause transition in. Risk factors for depressive symptoms during the perimenopause include prior standardized mean difference (MDD), psychosocial factors, anxiety symptoms, and reproductive-related mood disturbance. However, active vitamin D (VD), exerts protective and regulatory effects on the brain dopamine system and suggests that similar to the antidepressant. Therefore, serum 25(OH)D level may be negatively correlated with the perimenopausal depression.

**Methods:**

The study only selects clinical randomized controlled trials of depression in perimenopausal women. We will search each database from the built-in until October 2020. The English literature mainly searches Cochrane Library, PubMed, EMBASE, and Web of Science. While the Chinese literature comes from CNKI, CBM, VIP, and Wangfang database. Meanwhile, we will retrieve clinical trial registries and grey literature. Two researchers worked independently on literature selection, data extraction, and quality assessment. The dichotomous data is represented by relative risk, and the continuous is expressed by mean difference or standard mean difference, eventually the data is synthesized using a fixed effect model or a random effect model depending on the heterogeneity. The serum vitamin D level, Hamilton Depression Scale, or Beck Depression Inventory or Zung self-rating depression scale or patient health questionnare-9 were evaluated as the main outcomes. While several secondary outcomes were also evaluated in this study. The statistical analysis of this Meta-analysis was conducted by RevMan software version 5.3.

**Results:**

This meta-analysis will further determine the association analysis between VD level and depression in women perimenopause.

**Conclusion:**

This study determines the VD level is related to the occurrence of depression in perimenopausal women.

## 1 Introduction

In recent decades, the occurrence of hormone-related depression symptoms across the female lifecycle has gained much attention, and some scholars thought that the cause of this situation may be those women are particularly sensitive to changes of the hormonal in the postpartum and perimenopause.^[1,2,3]^

Perimenopausal depression is characterized by affective symptoms as well as menopause-specific somatic complaints.^[4]^ Many data manifested a particularly high prevalence of depressive symptoms in the peri or postmenopausal,^[4,5]^ and the perimenopause is a window of vulnerability for the development of both depressive symptoms and major depressive episodes, even in women with no history of major depressive disorder(MDD).^[1]^ Perimenopausal depression is a chronic recurrent disease with an increasing risk of suicide and self-mutilation.^[6–8]^ Depression not only does harm to people's health and affects their daily life, but also bring tremendous burden to their families and society.^[9]^ Risk factors for depressive symptoms during the perimenopause include prior MDD, sociodemographic factors, psychosocial factors, and reproductive-related mood disorder.^[5,9–11]^ Many researchers found that the decrease of ovarian estrogen production is a highly risk factor for depression,^[12]^ and vasomotor symptoms are positively related to it.^[13]^ Perimenopausal depression includes a wide range of symptoms,^[6]^ and several common symptoms of the perimenopause overlap with the symptoms of depression during this stage.^[1]^ Treatments for perimenopausal depression usually include antidepressants and hormone replacement therapy,^[14]^ and antidepressant treatment for perimenopausal depression usually begins with a selective serotonin reuptake inhibitor,^[6]^ non-hormonal and behavioral can also be deployed.^[15]^ Nowadays, more emphasis is on tailored management methods.

Vitamin D (VD) is a fat-soluble vitamin obtainable from the diet, which binds to the vitamin D receptor to enable its diverse physiological functions.^[16,17]^ The classical role of VD is to regulate metabolism of calcium and phosphate. However, a lot of research has suggested that VD deficiency is also associated with the increased risk of many other extra-skeletal diseases.^[18–20]^

The vitamin D receptors are widely distribution in human brain.^[21]^ Normal brain function depends on a fine balance between the activity of the excitatory and inhibitory neurons (E–I balance).^[22]^ VD may modify severity of brain dysfunction.^[21]^ In many neuropsychiatric illnesses, serotonin levels are low, and VD could regulate it,^[23]^ estrogens is also involved.^[10]^ In addition, VD could reduce inflammation, and the expression of DNA demethylases that controls the epigenetic landscape, thus enabling gene transcription to maintain normal neuronal activity and prevent depression.^[22]^ However, when VD levels decline, the levels of Ca^2+^ begin to rise within the cell and this may lead to the depression.^[22]^ There was a significant relationship between a low level of VD and postpartum depression among reproductive-aged Iranian women,^[24]^ and a study found significantly more depression in smokers compared to non-smokers, because the nicotine in cigarette might interfere with intestinal calcium absorption.^[25]^

In conclusion, this study will help to determine the relationship between serum VD level and perimenopausal depression. We hope this study will provide higher quality evidence for the association analysis between VD level and depression in women perimenopause.

## 2 Methods

### 2.1 Protocol registration

The systematic review protocol has been registered on the INPLASY website (https://inplasy.com/inplasy-2020-4-0117/) and INPLASY registration number is INPLASY202040117. It is reported following the guidelines of Cochrane Handbook for Systematic Reviews of Interventions and the Preferred Reporting Items for Systematic Reviews and Meta-analysis Protocol.^[26]^ If there are any adjustments throughout the study, we will fix and update the details in the final report.

### 2.2 Inclusion criteria

#### 2.2.1 Study design

The study only selects clinical randomized controlled trials (RCTs) of depression in perimenopausal women published in both Chinese and English. However, animal experiments, reviews, case reports, and non-RCT are excluded.

#### 2.2.2 Participants

The patients with clinically diagnosed perimenopausal depression, regardless of race, gender, and age. Patients with severe heart disease, liver, and kidney dysfunction, other mental illness, or a relevant drug allergic history will be not included.

#### 2.2.3 Interventions

The experiment group was women with depression in perimenopause with low serum VD, while the control group was with normal serum VD. In addition, the 2 groups did not take any drugs that interfered with the outcome indicators. The follow-up time was ≥12 weeks.

#### 2.2.4 Outcomes

The primary outcomes include *t* serum VD level, Hamilton depression scale or Beck depression inventory or Zung self-rating depression scale or patient health questionnare-9.

Additional outcome(s): Secondary outcomes are insomnia severity index or Pittsburgh sleep quality index or Hamilton anxiety scale, FSH levels and history of menstrual irregularity, serum estradiol measurements.

### 2.3 Search methods

#### 2.3.1 Electronic searches

Information sources: We will retrieve each database from the built-in until October 2020. The English literature mainly searches Cochrane Library, PubMed, EMBASE, and Web of Science. While the Chinese literature comes from CNKI, CBM, VIP, and Wangfang database. We adopt the combination of heading terms and free words as search strategy which is decided by all the reviewers. Search terms: serum vitamin D level, vitamin D, 25(OH)D, VD, 1,25(OH) 2D3, vitamin D deficiency, vitamin D supplementation, postmenopausal depression, depression in menopausal women, perimenopausal depression, menopause depression. We will simply present the search process of the Cochrane Library, as shown in Table 1 adjusting different search methods according to different Chinese and English databases.

**Table 1:**
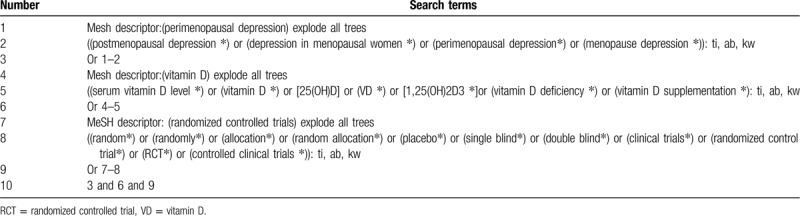
Cochrane Library search strategy.

#### 2.3.2 Searching other resources

At the same time, we will retrieve other resources to complete the deficiencies of the electronic databases, mainly searching for the clinical trial registries and gray literature about VD level and depression in women before and after menopause on the corresponding website.

### 2.4 Data collection and analysis

#### 2.4.1 Selection of studies

**Figure 1:**
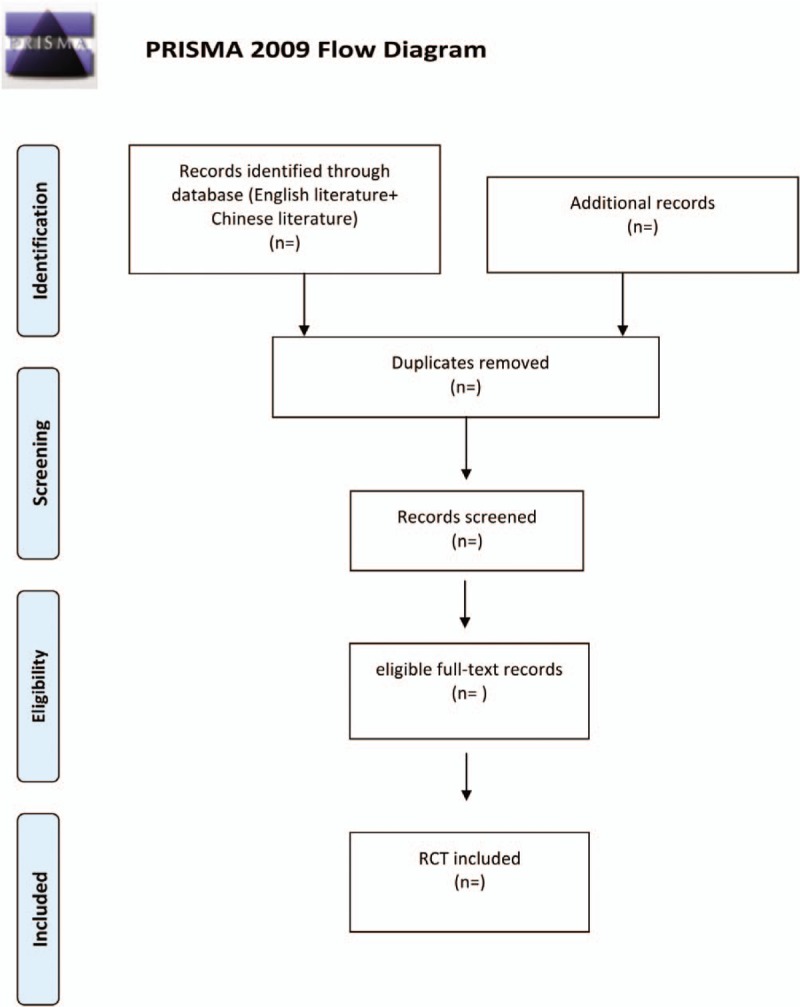
Flow chart of the study selection.

Import all literatures that meet the requirements into Endnote X8 software. First, 2 independent reviewers initially screened the literatures that did not meet the pre-established standards of the study by reading the title and abstract. Secondly, download the remaining literatures and read the full text carefully to further decide whether to include or not. Finally, the results were cross-checked repeatedly by reviewers. If there is a disagreement in the above process, we can reach an agreement by discussing between both reviewers or seek an opinion from third party. Preferred Reporting Items for Systematic Reviews and Meta-analysis flow diagram (Fig. 1) will be used to show the screening process of the study.

#### 2.4.2 Data extraction and management

According to the characteristics of the study, we prepare an Excel form for data collection before data extraction. Outcome indicators for eligible studies were independently extracted and filled in the data extraction form by 2 reviewers. If there is any argument, it can get an agreement by discussing through 2 reviewers or seek suggestions form third party. The main data extracted are as follows: title, author, year, fund source, sample size, age, gender, duration of disease, interventions, outcome measures, adverse reactions, etc. If you find something unclear in the study, you can contact the author of the communication directly for more detailed information. The above information was finally cross-checked by 2 reviewers.

#### 2.4.3 Assessment of risk of bias in included studies

The quality assessment of RCTs adopts the risk of bias assessment tool provided by the Cochrane Handbook. The following 7 items, such as random sequence generation, allocation concealment, blinding of participants, and personnel, blinding of outcome assessment, incomplete outcome data, selective outcome reporting, and other bias, are evaluated by 3 grades of “low bias”, “high bias” and “unclear bias”. The discrepancies will get a consistent conclusion by discussing between both reviewers or seeking the third-party consultation.

#### 2.4.4 Measures of treatment effect

Different evaluation methods are selected according to the different efficacy indicators. For the dichotomous data, we will choose the effect scale indicator relative risk with 95% CI to represent. While the continuous data is expressed as mean difference or standardized mean difference with 95% CI depending on whether the measurement scale is consistent or not.

#### 2.4.5 Dealing with missing data

The reviewers will contact the first author or correspondent author via email or telephone to obtain missing data if the relevant data is incomplete. If the missing data is still not obtained in the above way, we can synthesize the available data in the initial analysis. Furthermore, sensitivity analysis will be used to assess the potential impact of missing data on the overall results of the study.

#### 2.4.6 Assessment of heterogeneity

Heterogeneity will be assessed by Chi-squared test and I^2^ test. If I^2^ < 50%, *P* > .1, we consider that no statistical heterogeneity between each study and choose fixed effect model to synthesize the data. If I^2^ ≥ 50%, *P* < .1, indicating that there is a statistical heterogeneity, the data is integrated by the random effect model. In addition, due to differences in heterogeneity, we will conduct subgroup or sensitivity analysis to look for the potential causes.

#### 2.4.7 Data analysis

Review Manager software version 5.3 provided by the Cochrane Collaboration will be performed for data synthesis and analysis. The dichotomous data is represented by relative risk, continuous data is expressed by mean difference or standardized mean difference. If there is no heterogeneity (I^2^ < 50%, *P* > .1), the data is synthesized using a fixed effect model. Otherwise (I^2^≥50%, *P* < .1), a random effect model is used to analyze. Then subgroup analysis will be conducted basing on the different causes of heterogeneity. If a meta-analysis cannot be performed, it will be replaced by a general descriptive analysis.

#### 2.4.8 Subgroup analysis

If the results of the study are heterogeneous, we will conduct a subgroup analysis for different reasons. Heterogeneity is manifested in the following several aspects, such as race, age, gender, different intervention forms, pharmaceutical dosage, treatment course.

#### 2.4.9 Sensitivity analysis

Sensitivity analysis is mainly used to evaluate the robustness of the primary outcome measures. The method is that removing the low-level quality study 1 by 1 and then merge the data to assess the impact of sample size, study quality, statistical method, and missing data on results of meta-analysis.

#### 2.4.10 Grading the quality of evidence

In this systematic review, the quality of evidence for the entire study is assessed using the“Grades of Recommendations Assessment, Development and Evaluation” (GRADE) standard established by the World Health Organization and international organizations.^[27]^ To achieve transparency and simplification, the GRADE system divides the quality of evidence into 4 levels: high, medium, low, and very low.

## 3 Discussion

Depression has become an important factor affecting public health, and women suffer more often from depression than men, particularly in the perimenopausal stage.^[9]^ There was research revealed that there was no difference between early menopausal stage and late menopausal stage in depressive mood.^[9]^

Meanwhile, there has been a significant scientific interest in the relationship between VD status and depression. We speculate perimenopausal depression is negatively correlated with serum VD levels. Thus, we intend to collect RCTs about serum VD level for perimenopausal depression based on evidence-based medicine and conduct a meta-analysis of its efficacy to provide higher quality clinical evidence related to perimenopausal depression.

## Author contributions

**Conceptualization:** Jie Yuan, Tiantian Chen, Yuan Zhao

**Data curation:** Jie Yuan, Tiantian Chen, Yue Cao

**Formal analysis:** Shujun Wei, Penglong Yu

**Funding acquisition:** Jie Yuan, Yaling Lei

**Methodology:** Shujun Wei

**Project administration:** Yaling Lei, Jie Chen

**Resources:** Jie Yuan, Tiantian Chen, Penglong Yu

**Software:** Jie Yuan, Tiantian Chen

**Supervision:** Jie Chen

**Writing – original draft:** Jie Yuan, Tiantian Chen

**Writing - review and editing:** Yaling Lei
